# Heterogeneous nuclear ribonucleoprotein L facilitates recruitment of 53BP1 and BRCA1 at the DNA break sites induced by oxaliplatin in colorectal cancer

**DOI:** 10.1038/s41419-019-1784-x

**Published:** 2019-07-18

**Authors:** Wenjun Hu, Linping Lei, Xuqin Xie, Libin Huang, Qian Cui, Tang Dang, Gang Logan Liu, Yuan Li, Xiaofeng Sun, Zongguang Zhou

**Affiliations:** 1 Institute of Digestive Surgery, State Key Laboratory of Biotherapy and Cancer Center, West China Hospital, Sichuan University, 610041 Chengdu, Sichuan China; 2 The Clinical Hospital of Chengdu Brain Science Institute, MOE Key Lab for Neuroinformation, University of Electronic Science and Technology of China, 611731 Chengdu, Sichuan China; 3 School of Life Science and Technology, Huazhong University of Science and Technology, 430074 Wuhan, Hubei China; 4 Department of Oncology and Department of Clinical and Experimental Medicine, SE-581 83, Linköping University, Linköping, Sweden; 5 Department of Gastrointestinal Surgery, West China Hospital, Sichuan University, 37 Guo Xue Xiang, 610041 Chengdu, China

**Keywords:** Cancer therapeutic resistance, Double-strand DNA breaks

## Abstract

Although oxaliplatin is an effective chemotherapeutic drug for treatment of colorectal cancer (CRC), tumor cells can develop mechanisms to evade oxaliplatin-induced cell death and show high tolerance and acquired resistance to this drug. Heterogeneous nuclear ribonucleoprotein L (hnRNP L) has been proved to play a critical role in DNA repair during IgH class switch recombination (CSR) in B lymphocytes, while, its role in CRC and chemotherapeutic resistance remain unknown. Our study aims to uncover an unidentified mechanism of regulating DNA double-strand breaks (DSBs) by hnRNP L in CRC cells treated by oxaliplatin. In present study, we observed that knockdown of hnRNP L enhanced the level of DNA breakage and sensitivity of CRC cells to oxaliplatin. The expression of key DNA repair factors (BRCA1, 53BP1, and ATM) was unaffected by hnRNP L knockdown, thereby excluding the likelihood of hnRNP L mediation via mRNA regulation. Moreover, we observed that phosphorylation level of ATM changed oppositely to 53BP1 and BRCA1 in the CRC cells (SW620 and HCT116) which exhibit synergistic effect by oxaliplatin plus hnRNP L impairment. And similar phenomenon was observed in the foci formation of these critical repair factors. We also found that hnRNP L binds directly with these DNA repair factors through its RNA-recognition motifs (RRMs). Analysis of cell death indicated that the RRMs of hnRNP L are required for cell survival under incubation with oxaliplatin. In conclusion, hnRNP L is critical for the recruitment of the DNA repair factors in oxaliplatin-induced DSBs. Targeting hnRNP L is a promising new clinical approach that could enhance the effectiveness of current chemotherapeutic treatment in patients with resistance to oxaliplatin.

## Introduction

Colorectal cancer (CRC) is one of the most commonly diagnosed cancers in the world and is a leading cause of cancer-related mortality for both males and females^1^. However, despite the considerable advances of cancer therapy in recent years, surgery and chemotherapy are still the main approaches used for the treatment of CRC^2,3^. Adjuvant chemotherapy plays an important role in patients with stage III CRC and probably high-risk stage II colon cancer, whereas cytotoxic chemotherapy is the mainstay of treatment for patients with stage IV CRC^4–6^.

Oxaliplatin, a platinum-based anti-neoplastic drug, is one of the most effective chemotherapeutic drugs used for the treatment of CRC^2^. It exhibits high double-stranded DNA crosslinking activity, thereby impairing DNA replication and transcription^7–9^, eventually leading to substantial DNA double-strand breaks (DSBs) and cell apoptosis. Nevertheless, in many patients, cancer cells have been found to develop several mechanisms to evade oxaliplatin-induced cell death and show high tolerance and acquired resistance to this drug^10^. In this regard, it has been found that DSBs repair is one of the critical factors responsible for resistance to chemotherapy in many cancers^11,12^. Therefore, novel strategies designed to impair DNA repair may contribute to enhancing the chemosensitivity to oxaliplatin in CRC treatment.

Heterogeneous nuclear ribonucleoprotein L (hnRNP L) was originally defined as a RNA-binding protein containing four RNA-recognition motifs (RRMs), which specifically interacts with CA-repeat and CA-rich RNA elements^13^. It is one of a series of proteins that associate with heterogeneous nuclear RNAs (such as pre-mRNAs and mRNAs) and play major roles in the formation, packaging, and processing of mRNA^14^. Recently, several studies have shown that DNA break events can induce posttranslational modifications of certain hnRNP proteins, indicating that these proteins involved in RNA processing are a prominent group of factors that are regulated during the DNA damage response^15,16^. Moreover, a proportion of hnRNP proteins have been shown to localize to DNA damage sites^17^, indicating that some hnRNP proteins are recruited to DNA break sites to participate directly in the DNA repair process. hnRNP L is found to be associated with DNA repair in the class switch recombination (CSR) process of activated B cells^18^. CSR is initiated by activation-induced cytidine deaminase (AID)-induced cleavage of two DNA switch (S) regions, a donor and an acceptor locus, located 5′ to each C_H_ region^19^. The broken S regions are subsequently paired and recombined via the general repair mechanisms, namely, non-homologous end joining (NHEJ) or alternative end joining^20–23^. Depletion of hnRNP L is found to impair CSR by inhibiting the end joining process without altering DNA cleavage frequency in the S region^18^, indicating that hnRNP L is required for the DNA repair process.

In this study, to investigate the involvement of hnRNP L in the DNA repair of CRC cells, we performed siRNA silencing of hnRNP L and examined DSBs signals in different CRC cell lines treated with oxaliplatin. We found that knockdown of hnRNP L significantly enhanced DSBs signals and cell death in CRC cells. In addition, our finding that hnRNP L interacts with and modulate the phosphorylation level of DNA repair factors ATM, 53BP1 and BRCA1, indicating that hnRNP L is directly involved in the DNA repair process.

## Results

### HnRNP L is involved in the DNA damage response, including AID-induced CSR of B cells and genome instability of CRC cells

**Fig. 1:**
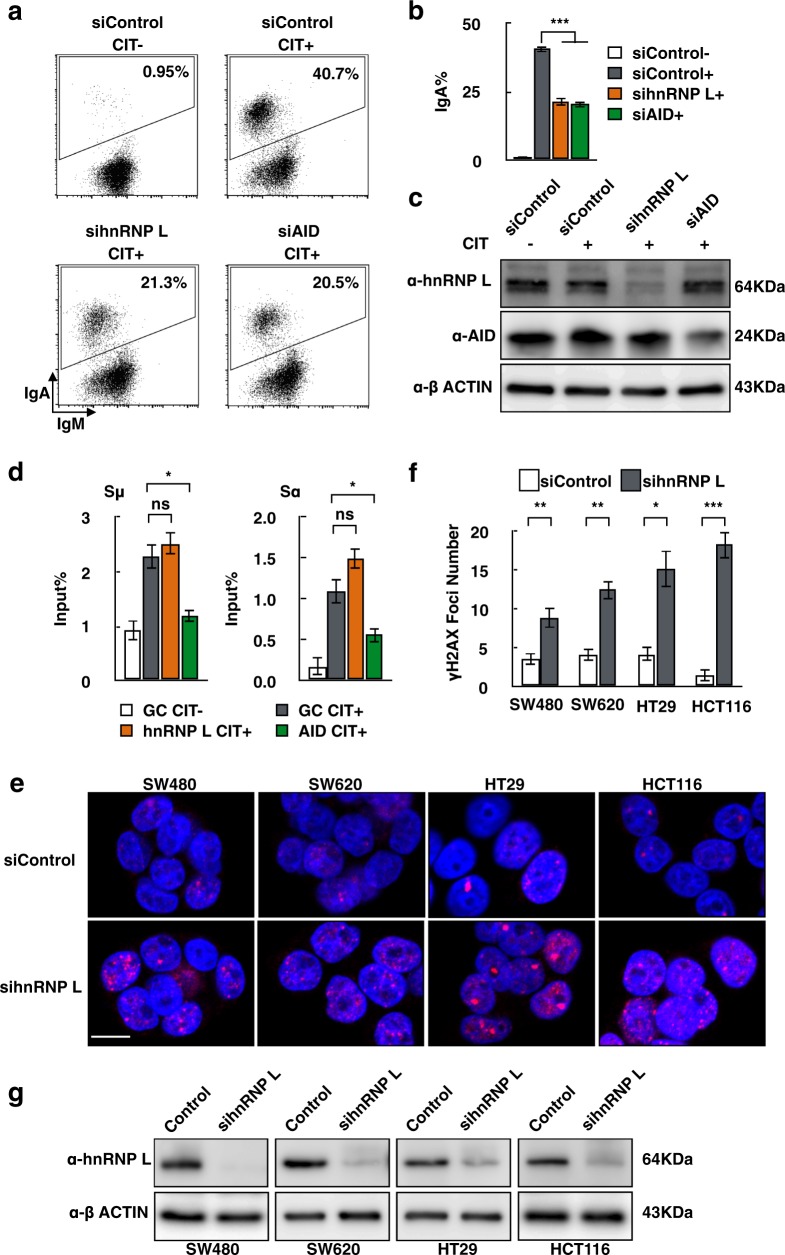
HnRNP L is found to be important for CSR recombination in CH12F3-2A cells and DNA-break level in CRC cells. **a** FACS profiles of IgA switching in CH12F3-2A cells transfected with hnRNP L and control siRNAs. Data are representative of three independent experiments, as shown in (**b**). The error bars represent the SEM; (+) and (−) represent the present and absence of CIT stimulation. SEM values were derived from three independent experiments. ***P < 0.001, unpaired *t* test. **c** Western blot analysis showing the knockdown efficiency of hnRNP L and AID. **d** DSBs determination by γH2AX ChIP assay using hnRNP L and control siRNAs in CH12F3-2A cells. The presence or absence of CIT stimulation is indicated by (+) or (−), respectively. SEM values were derived from three independent experiments. *P < 0.05, ns: no significant difference, unpaired *t* test. **e** Representative images of γH2AX staining in different human colorectal cancer cell lines treated with control hnRNP L siRNA. Nuclei were stained with DAPI. Scale bar represents 10 μm. **f** Histograms show the numbers of γH2AX foci per nucleus. Approximately 35–45 nuclei were evaluated for γ-H2AX foci formation for each sample, data are presented as mean ± s.e.m. *P < 0.05, **P < 0.01, ***P < 0.001, unpaired *t* test. **g** Western blot analysis showing the knockdown efficiency of hnRNP L in the indicated colorectal cancer cell lines

To examine the role of hnRNP L during CSR, we introduced RNAi oligonucleotides into CH12F3-2A cells to knockdown hnRNP L or AID (Fig. 1a). The FACS profiles showed that the knockdown of hnRNP L or AID significantly inhibited the IgA switching in CH12F3-2A cells stimulated with CIT (Fig. 1b). Western blotting confirmed that both proteins were significantly reduced following the introduction of the specific RNAi oligonucleotides (Fig. 1c). AID has been reported to be the most critical factor initiating DNA cleavage in the S region of the immunoglobulin locus during CSR^24^. To directly assess the requirement for hnRNP L in S-region DNA cleavage, we performed a γH2AX ChIP assay, which detects DSB-induced γH2AX focus formation in DNA regions flanking DSBs^18^. The depletion of AID, but not of hnRNP L, significantly reduced the γH2AX signal in the Sμ and Sα sequences, indicating that hnRNP L is more likely to be involved in the post-cleavage step of CSR (Fig. 1d).

A chemotherapy regimen containing oxaliplatin is the first-line treatment for CRC patients^25^. Oxaliplatin binds to DNA, introducing the formation of crosslinks and bulky adducts. The common enzymes for DNA repair in CRC cells and mouse B cells led us to postulate that hnRNP L may play a role in DSBs repair during chemotherapy. Next, we examined γH2AX foci in different CRC cell lines following treatment with hnRNP L siRNA (Fig. 1e–g). The results revealed that the signal of γH2AX foci in SW480, SW620, and HT29 cell lines increases after knockdown of hnRNP L, indicating that hnRNP L may function to protect DNA from breaks in these CRC cells.

### CRC cells show slight inhibition of proliferation by hnRNP L depletion

**Fig. 2:**
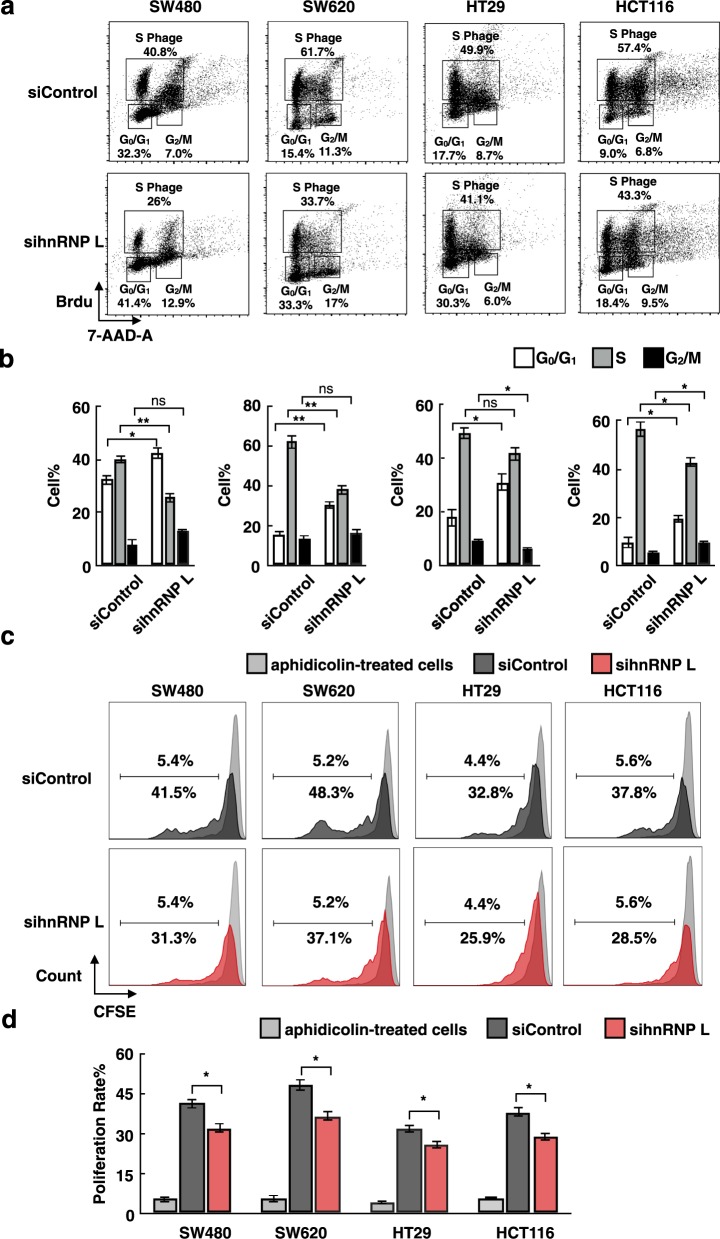
Knockdown of hnRNP L results in the slight inhibition of SW480, SW620, HT29, and HCT116 cell proliferation. **a** Cell cycle analysis using a BD BrdU FITC assay kit and flow cytometry were performed in colorectal cancer cell lines treated with control and hnRNP L siRNA. **b** Histograms show the percentages of cells in the G_0_/G_1_, G_2_/M, and S phases, mean ± SEM in triplicate experiments from various siRNA samples as indicated. *P < 0.05, **P < 0.01, ns: no significant difference, unpaired *t* test. **c** Cell proliferation after siRNA transfection was measured by FACS analysis of CFSE dilution at 48 h. The profile of aphidicolin-treated cells represents the cell proliferation arrest status (peaks indicate by the light gray color). These results are representative of three independent experiments. **d** Histograms show triplicate experiments using PI-cells derived from various siRNA samples as indicated. SEM values were derived from three independent experiments. *P < 0.05, unpaired *t* test

Prior to assessing the role of hnRNP L in DNA repair, we wanted to observe its effect on cell growth and proliferation. The thymidine analog BrdU is incorporated into newly synthesized DNA in cells entering and progressing through the S (DNA synthesis) phase of the cell cycle. The four cells lines treated with control siRNA and sihnRNP L were analyzed cytometrically at 48 h post-transfection (Fig. 2a). The percentage of cells in the S phase decreased in those cells with hnRNP L knockdown, whereas the percentage of cells in the G_0_/G_1_ phase was enhanced (Fig. 2b). These results showed that impairment of hnRNP L had a slight inhibitory effect on the cell cycle of CRC cells.

Next, we monitored and analyzed cell proliferation by CFSE staining. Cells treated with aphidicolin were used as a positive control for proliferation arrest (Fig. 2c). The results showed that cells with hnRNP L knockdown exhibited a slightly lower proliferation rate than those treated with control siRNA (Fig. 2d). Cells treated with aphidicolin showed only ~5% proliferation. This phenomenon is consistent with the findings of the cell cycle assay.

To demonstrate the functions of hnRNP L in colorectal cancer therapy, we were trying to employ CRISPR/Cas9 technology^26^ in SW620 cells and generated hnRNP L expression defective clones (see ‘Methods’ section for detail, and Supplementary Figure S1). Next, we utilized shRNA-mediated expression disruption by lentivirus vector to stable knockdown the expression level of hnRNP L (Supplementary Figure S2). We observed that inhibiting the expression of hnRNP L persistently and severely causes strong suppression on cell proliferation (Supplementary Figure S2, d), indicating that complete dysfunction of hnRNP L might be lethal to cells. So, siRNA knockdown of hnRNP L was used in most of the subsequent experiments.

### Colorectal cancer cells exhibited higher sensitive to oxaliplatin in the absence of hnRNP L

**Fig. 3:**
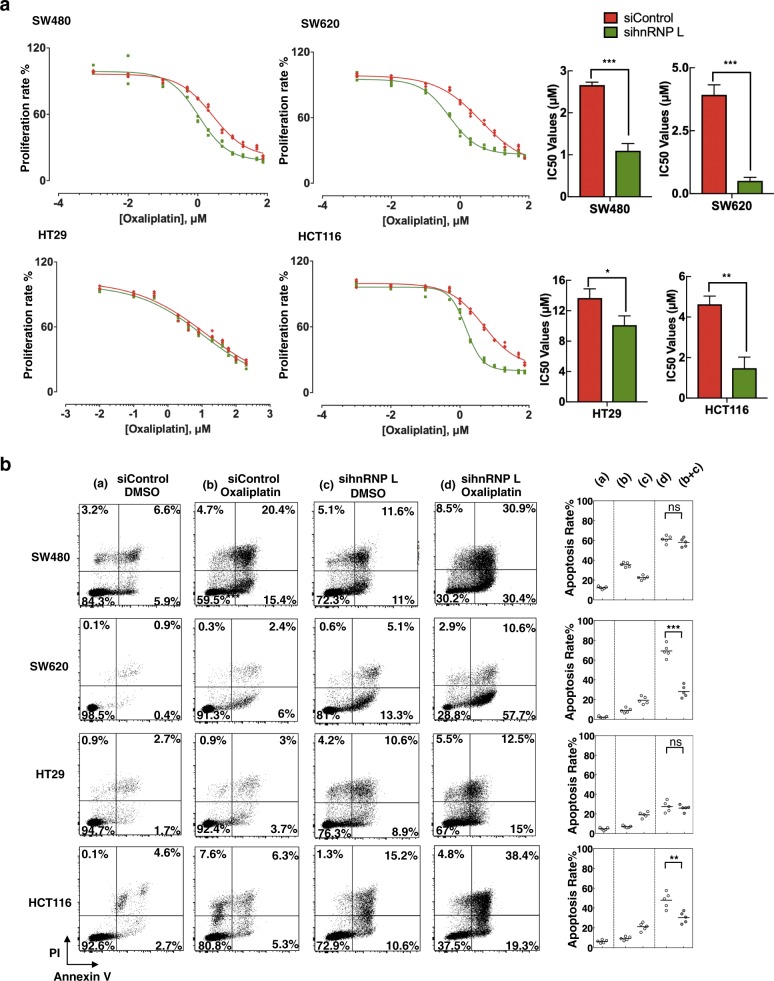
Absence of hnRNP L enhances the sensitivity of oxaliplatin in colorectal cancer cells. **a** CCK-8 assay was used to assess cell viability in colorectal cancer cells after treatment by increasing concentrations of oxaliplatin at 48 h with siControl or sihnRNP L siRNA. IC50 values of different colorectal cancer cell lines treated with siControl or sihnRNP L under oxaliplatin condition. IC50 of siControl vs. sihnRNP L, SW480 cells, 2.69 ± 0.15 vs. 1.07 ± 0.26, **P < 0.01; SW620 cells, 3.98 ± 0.29 vs. 0.50 ± 0.19, ***P < 0.001; HT29, 13.38 ± 2.1 vs. 10.25 ± 1.9, *P < 0.05; HCT116, 4.69 ± 0.84 vs. 1.52 ± 0.91, **P < 0.01. **b** Analysis of apoptosis by Annexin-V/PI double staining. Cells treated with sihnRNP L, cells treated with 15 µM oxaliplatin, and cells treated with both sihnRNP L and 15 µM oxaliplatin. Apoptosis data are shown as mean of quartic experiments. Statistical significance was evaluated by unpaired *t* test. **P < 0.01, ***P < 0.001, ns: no significant difference

As cancer cells are inhibited by oxaliplatin-induced DSBs and apoptosis^8^, we wanted to determine how the extent of DNA breakage would change if we impaired the ability of DNA repair via hnRNP L knockdown. The four cell lines examined in this study vary in appearance and growth characteristics (Supplementary Figure S3). Each cell line was treated with siControl or sihnRNP L siRNA, combined with or without 15 µM oxaliplatin, and we accordingly observed a significant reduction in cell distribution in the groups treated with both sihnRNP L siRNA and oxaliplatin. CCK-8 assays were performed with cells treated by siControl and sihnRNP L (Fig. 3a). IC50 of siControl vs. sihnRNP L, SW480 cells, 2.69 ± 0.15 vs. 1.07 ± 0.26, **P < 0.01; SW620 cells, 3.98 ± 0.29 vs. 0.50 ± 0.19, ***P < 0.001; HT29, 13.38 ± 2.1 vs. 10.25 ± 1.9, *P < 0.05; HCT116, 4.69 ± 0.84 vs. 1.52 ± 0.91, **P < 0.01.

Next, we quantitatively analyzed the total level of apoptosis using the four CRC cell lines (Fig. 3b). Cells treated with both sihnRNP L siRNA and oxaliplatin showed significantly higher levels of apoptosis than other groups in all the cell lines analyzed. Furthermore, we combined the cells death in the group of either sihnRNP L knockdown or oxaliplatin treatment and then compared with the double treated group. A synergistic effect of the combination of sihnRNP L siRNA and oxaliplatin could be found in SW620 and HCT116 cells, indicating that these two cell lines may be more sensitive to oxaliplatin under hnRNP L knockdown than other cells. And the changes of IC50 in SW620 and HCT116 cell lines were greater than SW480 and HT29 cells after hnRNP L knockdown, which were consistent with the results of apoptosis analysis.

### DSBs in colorectal cancer cells were significantly enhanced by hnRNP L knockdown when treated with oxaliplatin

**Fig. 4:**
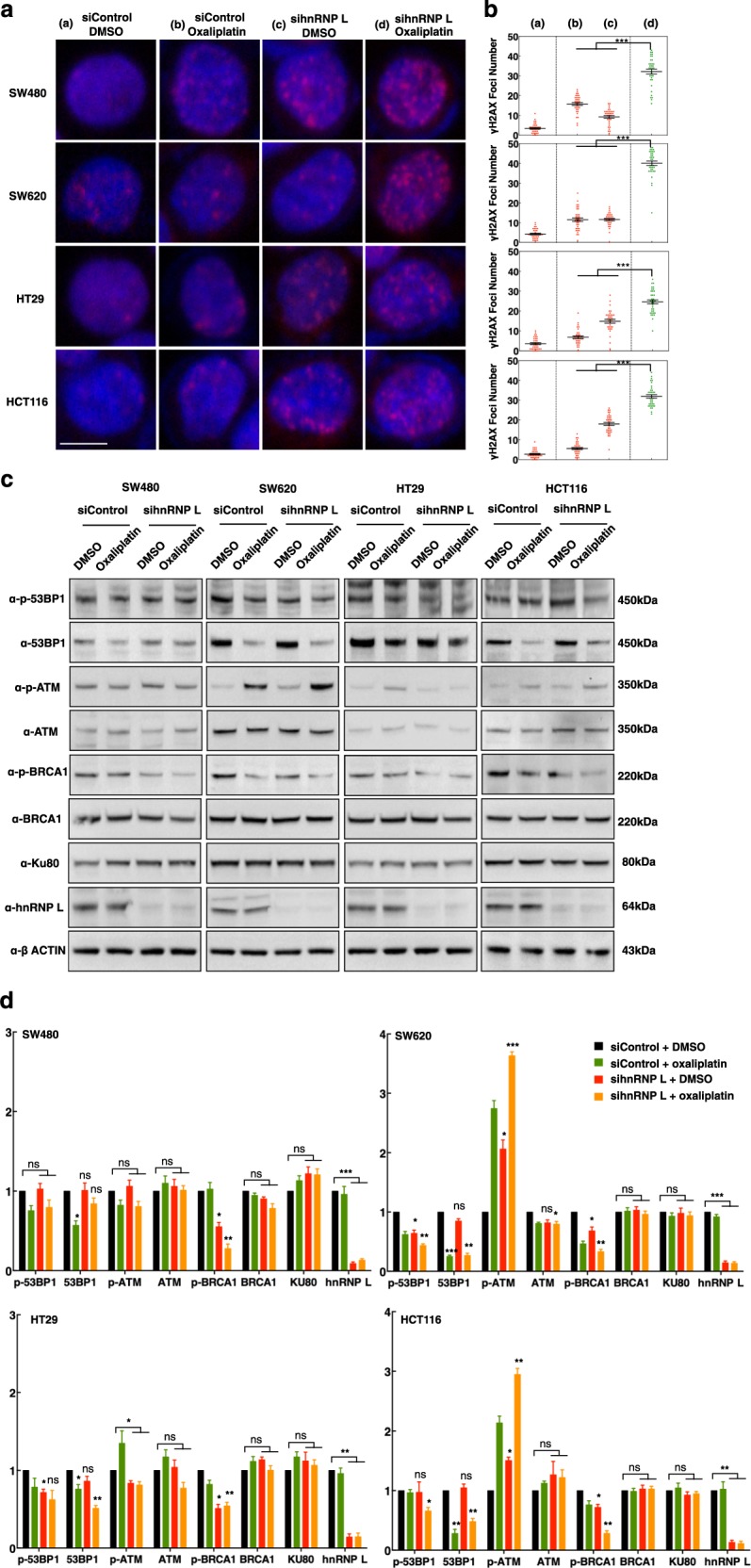
Deficiency of hnRNP L increases double-strand DNA breaks in response to treatment with oxaliplatin. **a** Representative images of γH2AX staining in different human colorectal cancer cell lines treated as indicated. The nuclei were stained with DAPI. Scale bar represents 10 μm. **b** Scatter dot plots show the numbers of γH2AX foci per nucleus. Approximately 35–45 nuclei were evaluated for γ-H2AX foci formation for each sample, data are presented as mean ± s.e.m. ***P < 0.001, unpaired *t* test. **c** Western blot analysis showing the expression and phosphorylation levels of critical DNA repair factors in the indicated colorectal cancer cell lines treated with sihnRNP L and/or oxaliplatin. **d** Quantitative analysis of the protein levels detected by western blotting. Data are presented as mean ± s.e.m. of three experiments. *P < 0.05, **P < 0.01, ***P < 0.001, ns: no significant difference, compared to siControl transfected cells incubated with DMSO, unpaired *t* test

To determine the mechanism whereby hnRNP L knockdown enhances apoptosis in CRC cells, we examined the level of DSBs by labeling γH2AX-positive cells (Fig. 4a). We observed that oxaliplatin significantly increased γH2AX formation. Knockdown of hnRNP L enhanced the γH2AX level in SW480, SW620, HT29 cells and HCT116 cells, which is consistent with the findings shown in Fig. 1d. Compared with other groups, the cells treated with both sihnRNP L oligo and oxaliplatin showed the strongest γH2AX signals in all four cell lines examined, indicating that DNA repair is impaired by hnRNP L knockdown (Fig. 4b).

### Phosphorylation level of ATM changed oppositely to 53BP1 and BRCA1 in the CRC cells which exhibit synergistic effect by oxaliplatin and hnRNP L impairment

DSBs are the most damaging form of DNA lesion, which can trigger genomic instability and lead to cell death if unrepaired^27^. These lesions are repaired by two major pathways: homologous recombination (HR) and NHEJ. HR is deployed in DSB repair in the S/G2 phase via a CDK-dependent process, whereas NHEJ occurs throughout the cell cycle in mammalian cells^28^. The type of DSB repair can also be determined by other factors, including the DNA-end structure of DSBs and the type of repair proteins binding to the break sites^29–31^. If NHEJ does not ensue when DSBs occur, repair switches to HR. BRCA1, is a key factor that plays multiple roles, including the control of DNA repair, and promotes HR by activating DNA ends^32^. In contrast, 53BP1, which is another critical player in DNA repair and signaling, inhibits excessive resection^33^. The ataxia-telangiectasia mutated (ATM) protein kinase is a master regulatory factor in the DSB response, which targets chromatin surrounding DSBs by phosphorylating S139 of histone variant H2AX to form γH2AX^34^. To elucidate how DNA damage repair is activated in oxaliplatin-treated CRC cells with or without hnRNP L knockdown, we examined and quantitative analyzed the protein expression and phosphorylation levels of ATM, BRCA1, 53BP1 and other reapair factors (Fig. 4c, d). We observed that p-ATM was significantly increased in SW620 and HCT116 cell lines, indicating that DSBs were still existing abundantly in these cells line. However, the significant lower levels of p-53BP1 and p-BRCA1 under sihnRNP L and oxaliplatin condition proclaimed deficiency of DNA repair in either NHEJ or HR pathway. It could be inferred that more DSBs were accumulated in these two cell lines, which explained synergistic effect by oxaliplatin and hnRNP L impairment. The p-ATM showed no significantly changed in SW480 and HT29 cell lines, indicating that DSBs in these cells were repaired by at least one of the pathways. SW480 and HT29 cells with sihnRNP L and oxaliplatin treatment exhibited lower level of p-BRCA1 but normal level of p-53BP1, which implied that these cells utilized NHEJ pathway to avoid genomic damages. The mRNA level of H2AX, ATM, BRCA1, and 53BP1 were examined and they were not affected by hnRNP L knockdown (Supplementary Figure S4). It seemed that the downstream activation step was blocked after ATM got phosphorylated and activated. It has been reported that both 53BP1 and BRCA1 are phosphorylated by ATM when they are recruit to the DSB sites^35^. We asked whether the location of these repair proteins was affected by hnRNP L impaired.

### HnRNP L is required for the foci formation of 53BP1 and BRCA1 at the DNA break sites induced by oxaliplatin

**Fig. 5:**
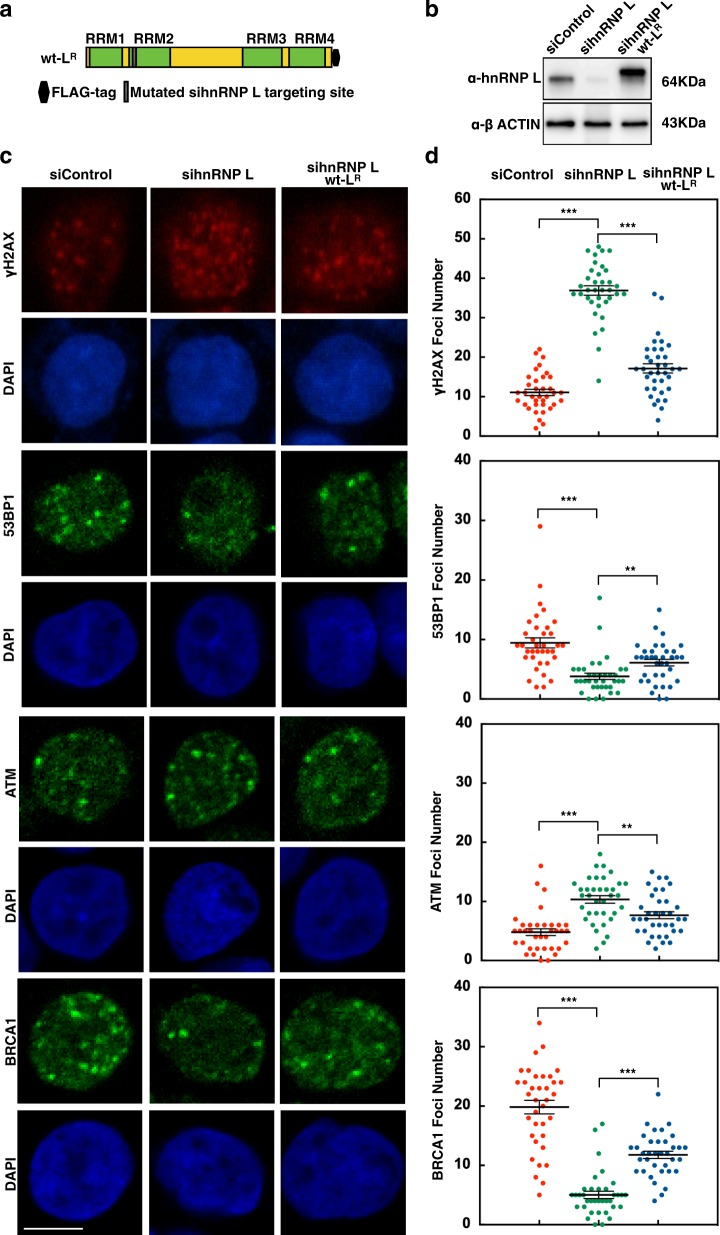
HnRNP L is critical for recruitment of 53BP1 and BRCA1 at the DNA break sites induced by oxaliplatin. **a** Representation of the siRNA-resistant hnRNP L mutants used in DNA repair factors foci complementation experiments. **b** Expression of exogenous hnRNP L-FLAG was confirmed by western blotting. **c** SW620 cells were treated as indicated and stained for γ-H2AX, 53BP1, ATM, and BRCA1 in order to reveal the recruitment of these DNA repair factors to the DNA break sites. Scale bar represents 10 μm. **d** Scatter dot plots show the numbers of γ-H2AX, 53BP1, ATM, and BRCA1 foci per nucleus. Approximately 35–45 nuclei were evaluated for foci formation for each sample, data are presented as mean ± s.e.m. **P < 0.01, ***P < 0.001, unpaired *t* test

To demonstrate unequivocally that hnRNP L is required for the recruitment of 53BP1 and BRCA1 to the DNA break sites, we introduced a construct expressing siRNA-resistant FLAG-tagged WT hnRNP L (wt-L^R^, where “R” denotes resistance to siRNA-mediated degradation) into SW620 cells (Fig. 5a, b). Then we used this system to monitor the changes of foci formation of ATM, γH2AX, 53BP1, and BRCA1 by modulating the expression level of hnRNP L (Fig. 5c). The foci formations were also examined in SW620 cells treated with siControl or sihnRNP L siRNA, combined with DMSO or 15 µM oxaliplatin (Supplementary Figure S5, a and b). We confirmed that depletion of hnRNP L impaired the foci formation of 53BP1 and BRCA1 while this phenomenon could be reversed by introducing exogenous hnRNP L. ATM exists as inactive dimers that dissociate and autophosphorylate on multiple residues when recruited to DSBs^36^. The activated ATM phosphorylates histone H2AX on Ser139 to promote downstream activation and then recruit 53BP1 and BRCA1^37^. The foci formation of ATM and γH2AX were enhanced when hnRNP L is impaired, and these foci were reduced after hnRNP L recovered. The results supported our interpretation that hnRNP L has a role downstream of ATM, γH2AX phosphorylation and is critical for the recruitment of 53BP1 and BRCA1.

### Either HR or NHEJ at I-Scel-induced DNA breaks was suppressed by hnRNP L

**Fig. 6:**
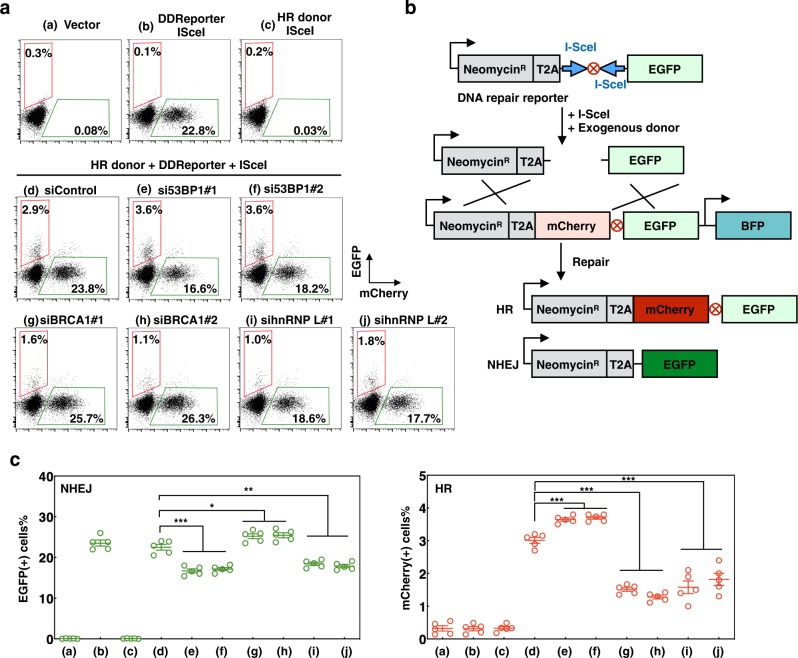
HnRNP L inhibits both HR and NHEJ at I-Scel-induced DNA breaks. **a** Representative flow cytometry for the DSB repair reporter. Ten thousand cells per sample were analyzed. **b** Schematics of the DRR consists of a promoter and resistance cassette fused to a T2A peptide and two inverted ISce1 sites, followed by GFP. An exogenous donor containing mCherry is conceived for HR. Repair by NHEJ or HR leads to GFP or mCherry expression, respectively. **c** Percentages of GFP + and mCherry + cells, gated on BFP +. Data are presented as mean ± s.e.m. of of five replicates, statistical significance was evaluated by unpaired *t* test. *P < 0.05, **P < 0.01, ***P < 0.001

Here we established an DSB Repair Reporter (DRR) assay in SW620 cells^38^. The hnRNP L knockdown cells used NHEJ significantly less frequently than the siControl cells (*P* < 0.01), and HR was significantly reduced in the same cells (*P* < 0.001) (Fig. 6a–c), indicating hnRNP L as the promotor of each pathway. 53BP1 and BRCA1 depletion by siRNA in SW620 cells were performed to conform the pathway choices of the system as they are the promoting factor of NHEJ and HR, respectively.

### The RNA-recognition motif (RRM) domains of hnRNP L are critical for its binding with DNA repair proteins

**Fig. 7:**
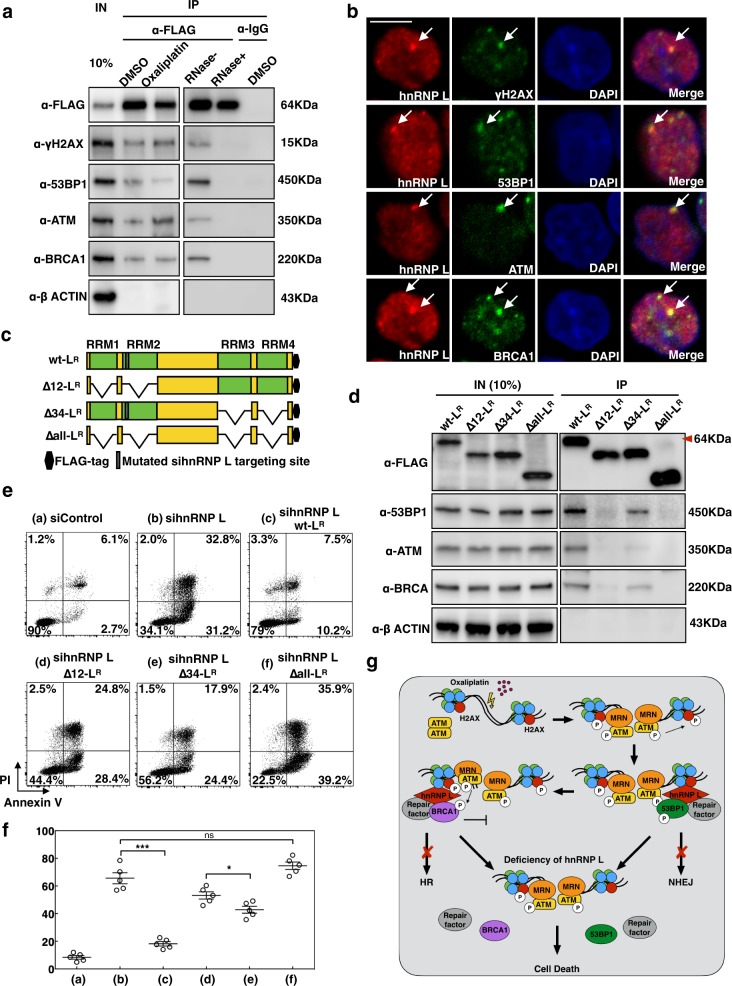
The RRM domains of hnRNP L are critical for its role in oxaliplatin-induced DNA repair. **a** Immunoprecipitation of exogenous hnRNP L-FLAG by anti-FLAG antibody, with or without RNAse. Cofactors were examined by western blotting. **b** HnRNP L colocalizes with DNA repair factors in oxaliplatin-treated SW620 cells, as demonstrated by immunofluorescent colocalization with γ-H2AX, 53BP1, ATM, and BRCA1. Images in red represent the detection by a Texas-red-conjugated secondary antibody, whereas green represents FITC. Nuclei were visualized by 4 Ј, 6 Ј -diamino-2-phenylindole staining. Scale bar represents 10 μm. **c** Representation of the various hnRNP L mutants used in apoptosis complementation and immunoprecipitation experiments. The “Δ” with numbers indicates the specific RRM domain deleted. **d** Western blot analysis shows the protein expression and interaction associated with each of the L_R_ constructs. **e** Representative fluorescence-activated cell sorter (FACS) data of apoptosis complementation experiments with different L_R_ constructs. **f** Apoptosis data are shown as mean ± s.e.m. of quartic experiments. Statistical significance was evaluated by unpaired *t* test. *P < 0.05, ***P < 0.001, ns: no significant difference. **g** Model for the role of hnRNP L in DNA repair caused by oxaliplatin-induced DNA breaks: hnRNP L binds with 53BP1 or BRCA1 and is recruited to DNA damage sites following the phosphorylation of ATM and H2AX. 53BP1 or BRCA1 fails to recruit to or retain at the break sites after hnRNP L depletion

As hnRNP L is critical for the recruitment of 53BP1 and BRCA1, we doubted whether hnRNP L forms complex(es) with these repair factors to play a direct role in DNA repair. Immunoprecipitation was utilized to pull down hnRNP L-FLAG and its cofactors by anti-FLAG antibody (Fig. 7a). We found that hnRNP L can bind with the repair proteins ATM, 53BP1, and BRCA1. FLAG-IP with RNAse were also performed and the results showed that bindings between hnRNP L with the DNA repair factors were disrupted completely, indicating that hnRNP L interacted with these DSBs repair proteins in RNA dependent manner. Colocalization experiments of hnRNP L with DNA repair factors in oxaliplatin-treated SW620 cells further demonstrated that hnRNP L interacted with γ-H2AX, 53BP1, ATM, and BRCA1 in oxaliplatin-induced DSBs (Fig. 7b).

Given that the RRM domains of hnRNP L are known to be critical for its activity^39^, in order to evaluate the importance of these domains in DNA repair, we constructed RRM-deleted hnRNP L mutants (Fig. 7c). All the mutants and WT constructs were modified so that they were resistant to the hnRNP L-targeted siRNA and were tagged at the C terminus with a FLAG epitope. Immunoprecipitation was performed using an anti-FLAG antibody to detect the interactions among hnRNP L mutants and DNA repair factors (Fig. 7d). We found that the RRM domains of hnRNP L were critical for the binding with these DNA repair proteins and that RRM1 and RRM2 play more important role than other RRMs. We next examined their apoptosis complementation efficiencies in SW620 cells treated with hnRNP L siRNA and oxaliplatin (Fig. 7e). Mutants lacking two of the RRM domains were partially defective in the apoptosis rescue function, and the mutant devoid of all four RRMs (Δall-L^R^) lost most rescue ability. The mutant with RRM1 and RRM2 deletion (Δ12-L^R^) caused more cell death than Δ34-L^R^ mutant, suggesting that RRM1 and RRM2 have a more important role in both proteins interacting and cell survival. We also found that ∆all-L^R^ could hardly rescue the foci formation of 53BP1 and BRCA1 while this phenomenon could be partially reversed by introducing ∆12-L^R^ or ∆34-L^R^ plasmid (Supplementary Figure S6, a and b). The foci formation of ATM and γH2AX exhibited opposite tendency compared to 53BP1 and BRCA1, which is consistent with the conclusions in Fig. 7c, e.

## Discussion

In the present study, we demonstrated the direct involvement and promoting role of hnRNP L in DNA repair for DSBs in CRC cells. This function of hnRNP L is inferred to be universal since enhanced DSB signals were observed in both CIT-stimulated CH12F3-2A cells and in oxaliplatin-treated CRC cells with depleted hnRNP L. We demonstrated that knockdown of hnRNP L significantly increased the sensitivity of all four examined CRC cell lines to oxaliplatin. Moreover, we observed the synergistic effect of combined hnRNP L depletion and oxaliplatin treatment on the mortality of SW620 and HCT116 cells. The high level of p-ATM and low levels of p-53BP1 and p-BRCA1 under sihnRNP L and oxaliplatin treatment (Fig. 4d), indicating that DSBs repair signaling was impacted in these two cell lines.

Oxaliplatin used in conjunction with folinic acid and 5-fluorouracil (FOLFOX) is widely used through intravenous injection to treat mainly stage II and III CRC patients after surgery^40^. It has been reported that 50% of CRC patients receiving FOLFOX develop drug resistance at a later stage of treatment, thereby resulting in a high probability of cancer recurrence and metastasis^5,40,41^. DSBs induced by oxaliplatin cause fatal damage to CRC cells. These breaks are assumed to be the most dangerous type of lesions in cells because they severely impair DNA replication and RNA transcription, and cause chromosomal translocations^42^.

An antagonistic relationship between BRCA1 and 53BP1 has been shown to promote different pathways for the repair of DSBs. NHEJ is considered to be the initial repair response, as Ku70/80 heterodimers bind rapidly to DSBs^31,43^. BRCA1 promotes 53BP1 dephosphorylation and RIF1 release to direct the repair pathway to HR^44^. In contrast, 53BP1 forms a barrier that prevents HR by inhibiting excessive resection^33^. ATM activates DSB repair through phosphorylation of downstream proteins, such as H2AX. It has been reported that ATM also phosphorylates 53BP1 at multiple sites to facilitate recruitment of numerous 53BP1-interacting proteins that are required for DSB repair^35^. In the present study, we found that the expression levels of BRCA1, 53BP1, and ATM were unaffected by hnRNP L knockdown, thereby indicating that hnRNP L does not function via transcriptional regulation. Moreover, we found the phenomenon that phosphorylated ATM was significantly enhanced while both phosphorylation level of 53BP1 and BRCA1 were reduced in the CRC cell lines which showed synergistic effect by oxaliplatin and hnRNP L depletion. We further confirmed the foci formation of ATM, γH2AX, 53BP1, BRCA1 by complementation experiments and found they are correlated with the phosphorylation level. The DDR assay showed hnRNP L knockdown cells used NHEJ significantly less frequently than the siControl cells, and HR was significantly reduced in the same cells, indicating hnRNP L as the promotor of each pathway. All the results supported our interpretation that hnRNP L has a role downstream of ATM, γH2AX phosphorylation and is critical for the recruitment of 53BP1 and BRCA1.

Immunoprecipitation and colocalization experiments both demonstrated that hnRNP L interacted with γ-H2AX, 53BP1, ATM, and BRCA1. As the RRMs of hnRNP L are the main functional domains, we constructed different RRM domain-deleted hnRNP L mutants to evaluate their importance in protein binding and function. Mutants lacking RRM1 + RRM2 or RRM3 + RRM4 were partially defective in cell death rescue function, and the mutant devoid of all four RRMs (Δall-LR) lost the most cell death rescue ability. And the foci formation of DNA repair factors also showed relevant changes. These findings suggest that the RRMs (RRM1 and RRM2, especially) play a critical role in the DNA repair activity of hnRNP L. The RRM domains of hnRNP L are known to be responsible for its RNA-binding activity, and an important role for RNA molecules in DNA repair has been suggested. Recently, an RNA-templated repair mechanism has been detected in both yeast and human cells, the latter of which utilize NHEJ machinery^45,46^. It has also been shown that R-loops, formed by hybrid RNA/DNA, are functionally important intermediate regulatory structures involved in DSB and DNA repair^47^. All these strands of evidence are consistent with a deduction that hnRNP L may function as a platform to connect the relevant RNAs and proteins to accomplish DNA repair processes via either HR or NHEJ. And the result of FLAG-IP with RNase further confirmed our speculation. In conclusion, we postulate that hnRNP L binds with 53BP1 or BRCA1 and is recruited to DNA damage sites following the phosphorylation of ATM and H2AX. 53BP1 or BRCA1 fails recruiting to retaining at the break sites after hnRNP L depletion (Fig. 7g). Our results provide a rationale for further investigations that focus on enhancing the sensitivity of cells to chemotherapeutic agents, including oxaliplatin, as well providing new insights for RNA-related DNA repair research. Thus, targeting hnRNP L could directly contribute to advances in the treatment of CRC.

## Methods

### Cell culture

The colorectal cancer cell lines SW480, SW620, HCT116, and HT29 cells were cultured in a DMEM medium (Invitrogen) containing 10% (vol/vol) FBS and penicillin–streptomycin. CH12F3-2A cells were cultured in RPMI 1640 medium (Invitrogen) supplemented with 10% (vol/vol) FBS.

### CSR assay and siRNA oligonucleotide transfection

CH12F3-2A cells were stimulated by CIT (CD40L, TGF-β, and IL-4) to induce class switching as previously described^18^. siRNA oligonucleotides (Supplementary Table S1, Invitrogen) were introduced into the cells by electroporation (Amaxa). The transfected cells were cultured for 24 h before the addition of CIT and subjected to fluorescence-activated cell sorter (FACS) analysis after 24 h of CIT stimulation. FITC-conjugated anti-IgA (eBioscience) and PE-conjugated anti-IgM (eBioscience) antibodies were used for surface IgM and IgA staining, respectively.

### CRISPR/Cas9-mediated gene disruption and shRNA-mediated expression blocking

To design the guide RNAs (gRNAs) for hnRNP gene targeting, a software tool (https://www.crisprscan.org/) predicting unique target sites throughout the human genome was used. Two oligonucleotides designed for target sites (Supplementary Table S1) were annealed and cloned into the linearized GeneArt CRISPR Nuclease CD4 Reporter Vector (CRISPR Nuclease Vector, Invitrogen). In the case of L22 clone, only one of the hnRNP L alleles was disrupted, while other clones showed no alleles damaged (Supplementary Figure S1, a and b). Then we performed another round of CRSPR on the clone L22, however, no alleles disrupted clones could be found in the subclone screening (Supplementary Figure S1, c and d).

The target sequencing containing shRNA vectors were provided by GENECHEM (Supplementary Figure S2). Plasmid DNA was transfected into 293T cells by mixing with FuGENE6 (Promega) and PCL Ampho (Solarbio). Collected the supernatant medium of 293T after 48 h and incubated SW620 cells for 5 h. Changed to fresh medium and cultured the cells for another 3 days. Transfected SW620 cells were then subjected to puromycin to obtain shRNA high expression cells (GFP containing).

### Plasmid construction

To generate hnRNP L-3 × FLAG fusion constructs, human hnRNP L (NM_001005335) was amplified by RT-PCR and cloned into a pCMV6Entry vector (Origene). To generate siRNA-resistant constructs, the siRNA-targeting sequences in hnRNP L were modified (Supplementary Table S1).

### Protein extraction and western blotting

Cells were harvested and washed with PBS before being lysed with cell lysis buffer (CST). The amounts of extracted proteins were determined using the BCA assay method (ThermoFisher). Briefly, 10 μg of each protein was electrophoresed on 4–20% SDS-PAGE gels (ThermoFisher), transferred to polyvinylidene difluoride (PVDF) membranes (ThermoFisher), and then blocked with 5% BSA at room temperature for 1 h. The membranes were then incubated overnight at 4 °C with the primary antibody (1:2000, Supplementary Table S2). After washing, the membranes were incubated with secondary antibody (1:2000, CST) at room temperature for 1 h.

### Chromatin immunoprecipitation (ChIP) assay

The ChIP assay was performed using an ActiveMotif ChIP-IT Express Kit according to the manufacturer’s instructions. In brief, 5 × 10^6^ cells were fixed in the presence of 1% formaldehyde for 5 min at room temperature. The reaction was stopped by the addition of 0.125 M glycine. A soluble chromatin fraction containing fragmented DNA of 500–2000 bp was obtained after cell lysis and sonication. ChIP was performed by incubating the cleared lysate with 3 μg anti-histone gamma-H2AX (γH2AX) antibody. IPed DNA was analyzed by real-time PCR, with the data initially being first normalized to the amount of input followed by normalization to the maximum value in each data set, as described previously^18^.

### Immunocytofluorescence assay

Cells were fixed with 4% paraformaldehyde for 20 min, permeabilized with 0.2% Triton X-100 for 5 min, and then incubated with primary and secondary antibodies. Nuclei were stained with DAPI diluent (300 nM; Sigma) at room temperature for 5 min. Localization of γH2AX was determined by fluorescence microscopy (Olympus, Tokyo, Japan) using 590 nm (Alexa-fluor 594) and 358 nm (DAPI) excitation wavelengths.

### BrdU-PI cell cycle assay (BrdU Flow kit, BD, cat.559619)

Cells were seeded in 6-cm dishes and incubated for 24 h to facilitate attachment. Subsequently, the cells were incubated for 8 h with 10 μM bromodeoxyuridine (BrdU) in growth medium, trypsinized, washed in PBS, and fixed in BD Cytofix/Cytoperm Buffer for 30 min. Fixed cells were washed in 1 × BD Perm/Wash buffer, resuspended in BD Cytoperm Permeabilization Buffer Plus, and incubated for 10 min on ice. Thereafter, the cells were washed with 1 × BD Perm/Wash buffer, resuspend in 100 µL Cytofix/Cytoperm Buffer for 5 min, washed once again with 1 × BD Perm/Wash buffer, and then treated with diluted DNase (30 µg of DNase/10^6^ cells). Subsequently, anti-BrdU-FITC antibody (20 min, room temperature) was added after washing. Following incubation, the cells were washed with 1 mL of 1 × BD Perm/Wash buffer and resuspended in 20 µL of the 7-aminoactinomycin (7-AAD) solution for 5 min. Finally, 500 µL of staining buffer was added to the cell suspension for FACS analysis.

### Cell proliferation assay

The cells were spread and cultured for 24 h then labeled with carboxyfluorescein succinimidyl ester (CFSE, Invitrogen, 5 μM) for 15 min at 37 °C. CFSE, which labels long-lived intracellular molecules with a fluorescent dye, was used to monitor cell proliferation status along with the standard cell counting. Portions of cells were treated separately with aphidicolin (2 μg/mL), a routinely used inhibitor of cell-cycle progression, which served as a positive control for proliferation arrest.

### Apoptosis assay

Cells treated with oxaliplatin were harvested after 24 h, digested, washed, gently suspended with 200 μL combining solution, and then gently mixed with 2 μL Annexin-V-FITC and 2 μL PI (BD Biosciences). Thereafter, the cells were incubated in the dark for 10 min at room temperature (20–25 °C), followed by the addition of 200 μL washing buffer. Approximately 10,000 cells from each sample were subjected to flow cytometry (Beckman), with FlowJo software being employed to analyze the data.

### DSB Repair Reporter assay

pLCN DSB Repair Reporter (DRR) and pCAGGS DRR mCherry Donor EF1a BFP were gifts from Jan Karlseder (Addgene plasmid # 98895; Addgene plasmid # 98896). The integrated DSB repair reporter (DRR), consists of a promoter and resistance cassette fused to a T2A peptide and two inverted ISce1 sites, followed by GFP. Intact or partially cut DRRs lack GFP expression owing to the presence of a STOP codon. SW620 cells were transfected with ISce1 and an exogenous donor for HR. Repair by NHEJ or HR leads to GFP or mCherry expression, respectively.

### CCK8

Cells (0.5 × 10^6^) were transiently transfected with siControl or sihnRNP L and cultured in 10-cm dish for 48 h. Live cells were digested and seeded to the 96-well plate (5000 cells/well, 12 × 3 array) and cultured for 24 h. Oxaliplatin was added with different concentration as shown (Fig. 3a). Discard the supernatant of cells 48 h later, add 100 μL of 10 times dilution CCK (Absin) into each well and incubated at 37 ℃ for 2 h. Test the OD value by a microreader with 450 nm light. Survival rate (%) = (OD_oxaliplatin_−OD_background_)/(OD_Control_−OD_background_).

### Immunoprecipitation

Cells (1.5 × 10^6^) were transiently transfected with 4 μg of a 3 × FLAG-tagged human hnRNP L construct. Live cells were lysed in 200 μL RNA-binding protein immunoprecipitation (RIP) lysis buffer (Millipore) 48 h later. The FLAG-tagged proteins in 50 μL of lysate were immunoprecipitated with 5 μg anti-FLAG antibody (Sigma) bound to Protein G magnetic beads. The beads were washed and resuspended in RIP Wash buffer (Millipore) and the protein–RNA complexes were eluted with a 0.2 M glycine solution.

### Statistical analysis

Statistical analysis was conducted with the two-tailed unpaired Student’s *t* test. All data represent the mean ± s.e.m. of three or more independent experiments.

## Supplementary information

Supplementary Figure S1

Supplementary Figure S2

Supplementary Figure S3

Supplementary Figure S4

Supplementary Figure S5

Supplementary Figure S6-1

Supplementary Figure S6-2

Supplementary Table S1

Supplementary Table S2

Supplementary figure legends
