# Supplementary figures and images for: Heterogeneous nuclear ribonucleoprotein L facilitates recruitment of 53BP1 and BRCA1 at the DNA break sites induced by oxaliplatin in colorectal cancer
Source: Cell Death Dis. 2019 Jul 18;10(8):550. doi: 10.1038/s41419-019-1784-x (PMC6639419; doi:10.1038/s41419-019-1784-x)

Figure.S1

**a**

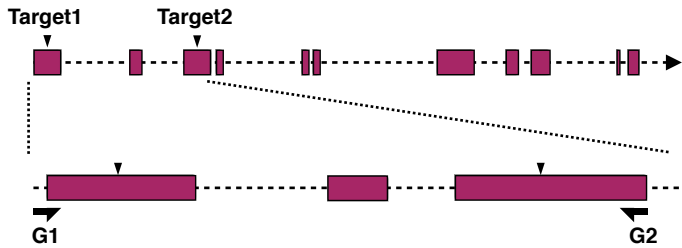

**b**

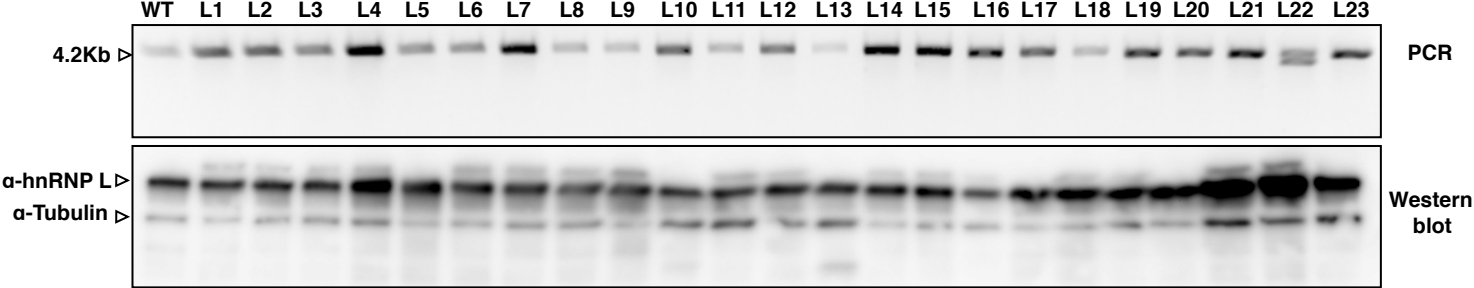

**c**

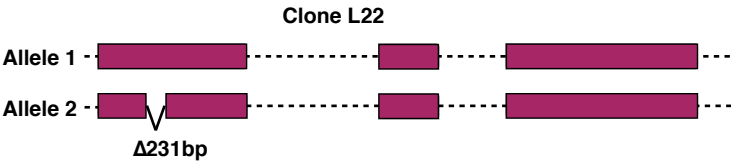

**d**

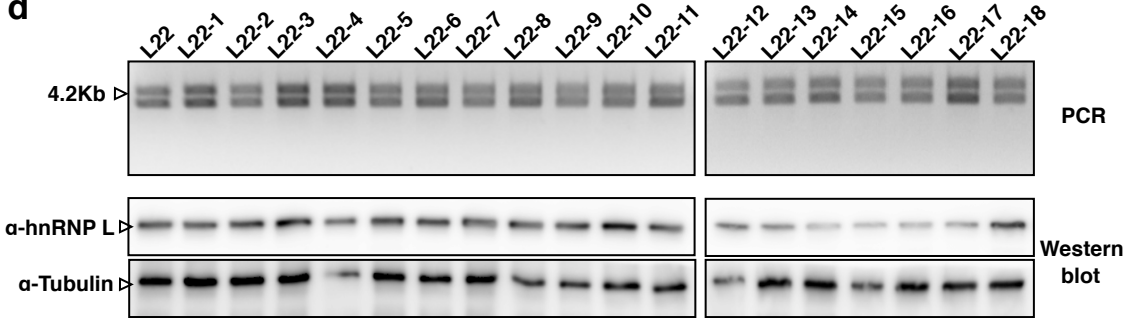

**e**

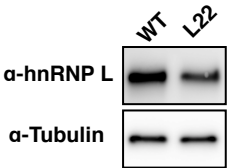

Supplement: Supplementary file 1 — Supplementary Figure S1 [file 41419_2019_1784_MOESM1_ESM.pdf]

Figure.S2

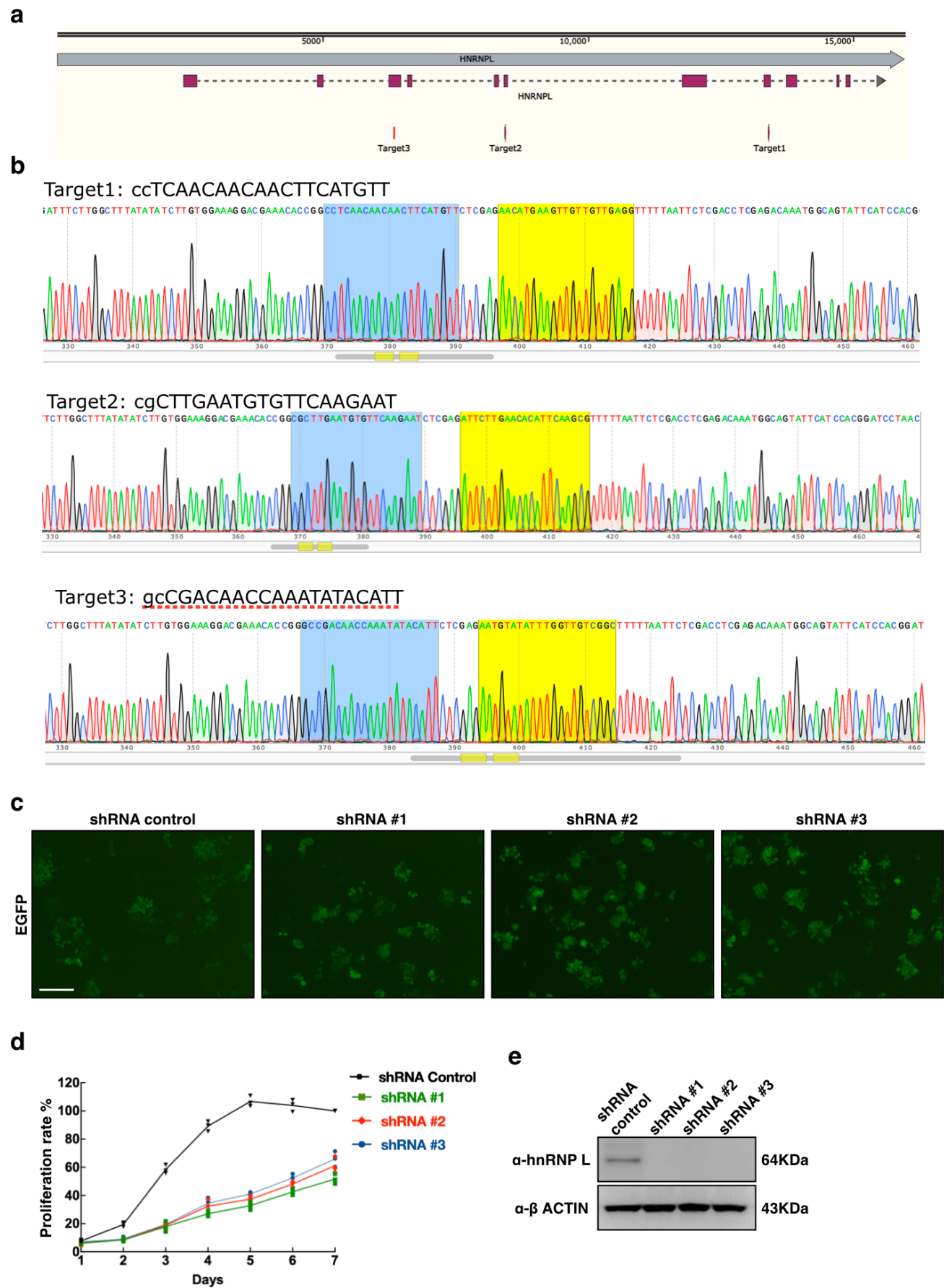

Supplement: Supplementary file 2 — Supplementary Figure S2 [file 41419_2019_1784_MOESM2_ESM.pdf]

Figure.S3

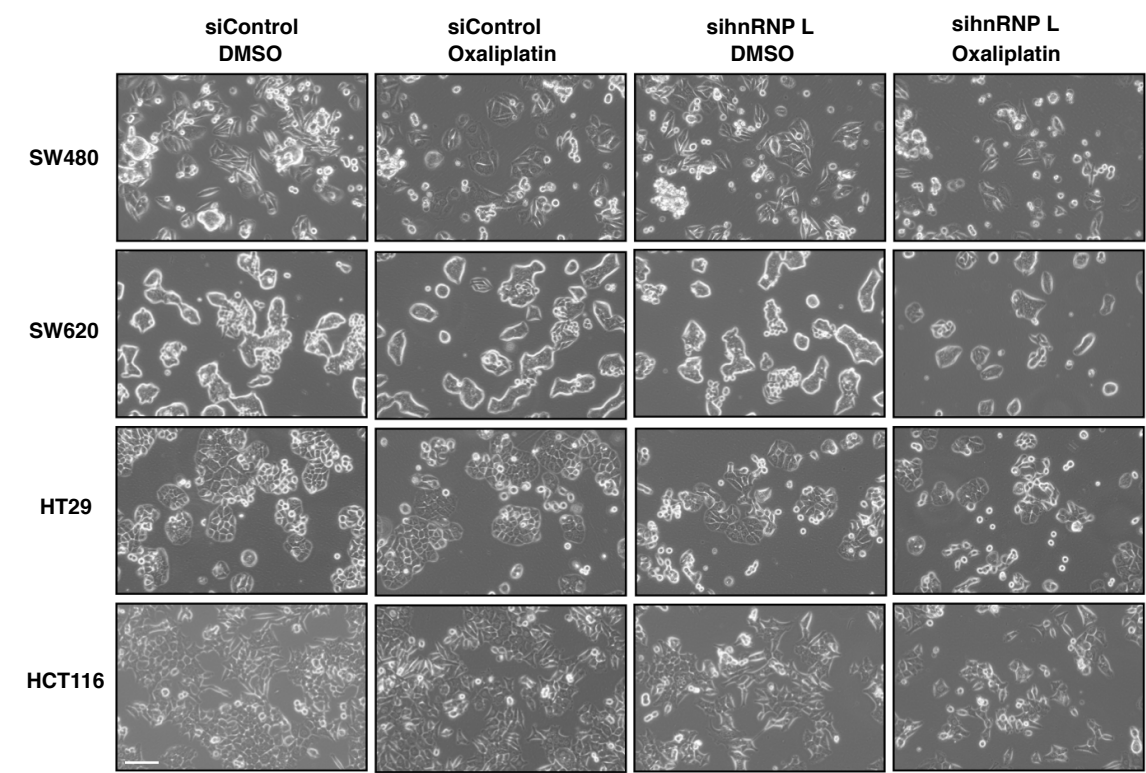

Supplement: Supplementary file 3 — Supplementary Figure S3 [file 41419_2019_1784_MOESM3_ESM.pdf]

Figure.S4

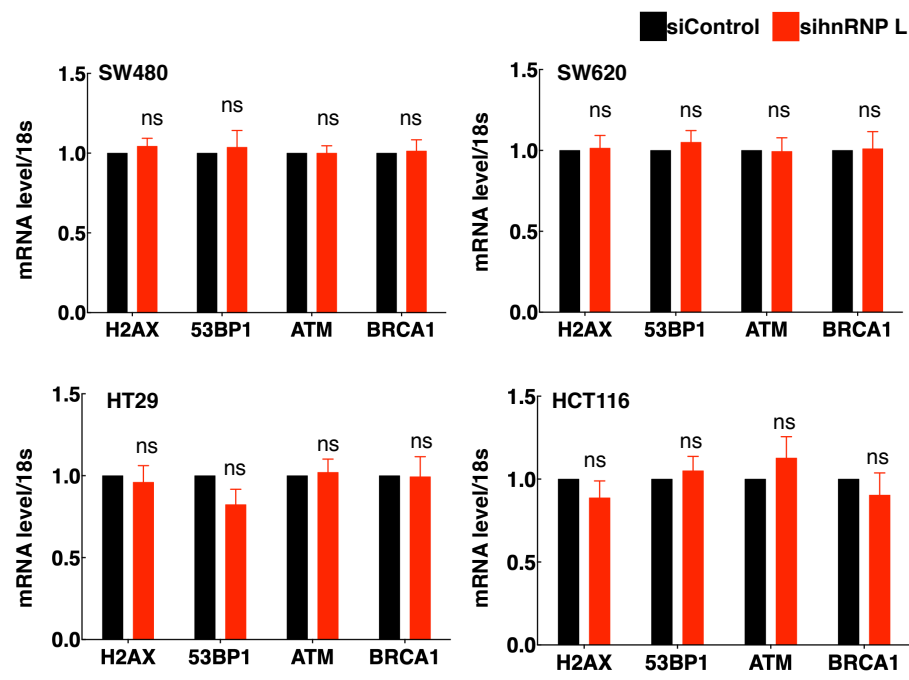

Supplement: Supplementary file 4 — Supplementary Figure S4 [file 41419_2019_1784_MOESM4_ESM.pdf]

Figure.S5

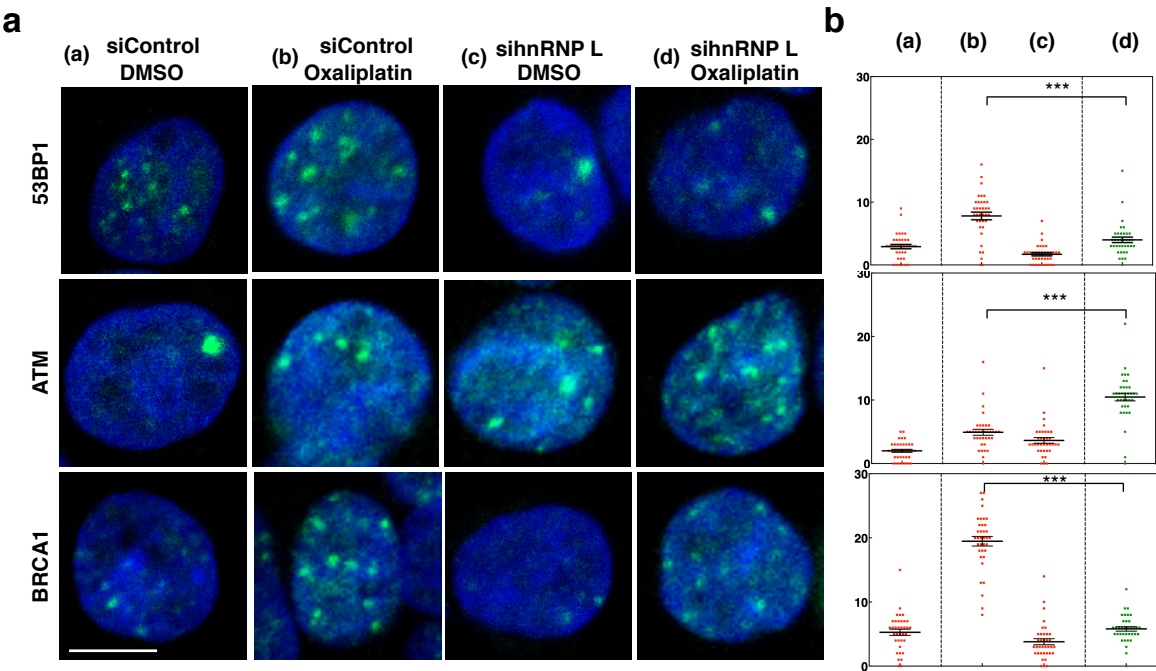

Supplement: Supplementary file 5 — Supplementary Figure S5 [file 41419_2019_1784_MOESM5_ESM.pdf]

Figure.S6

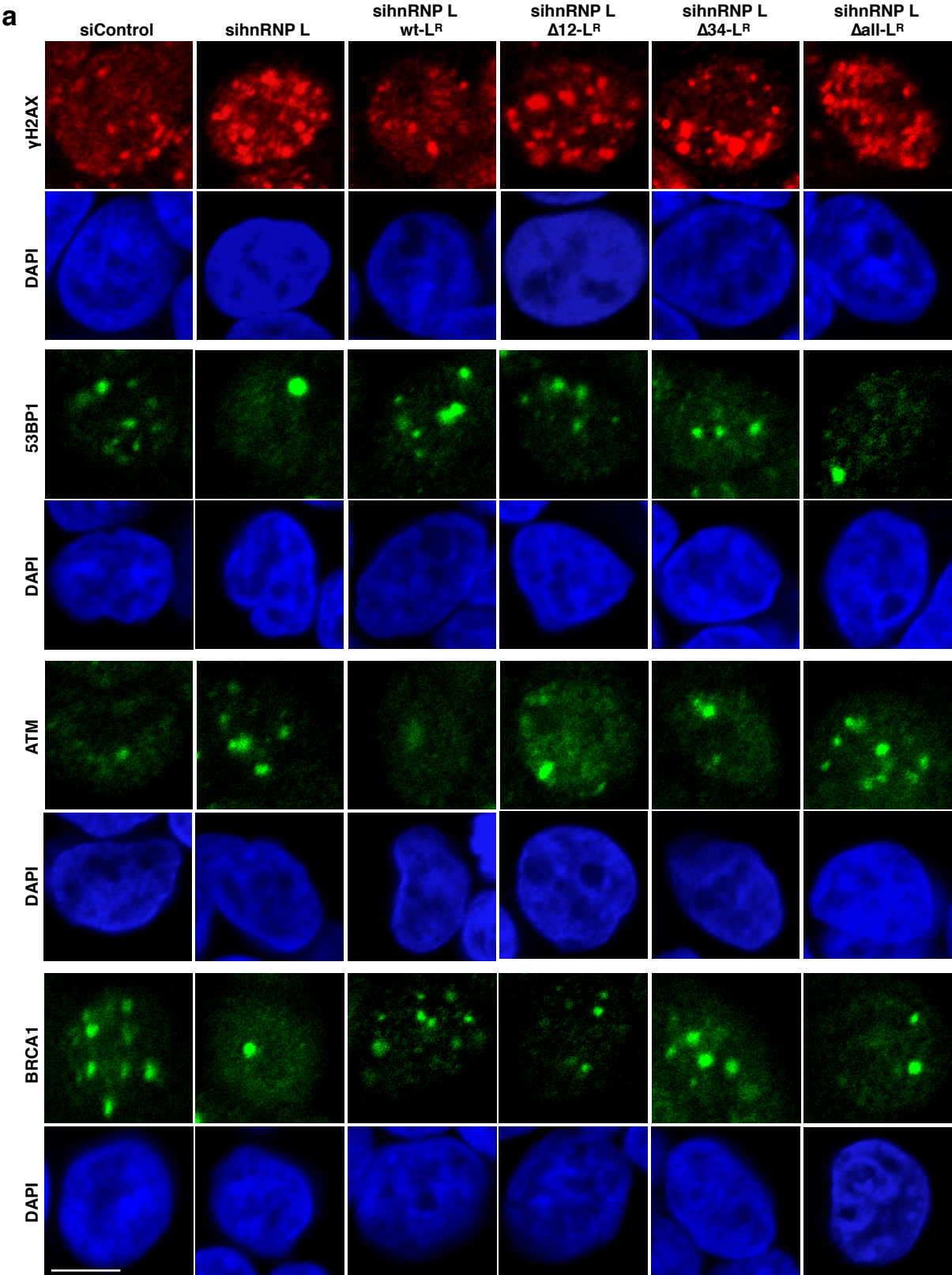

Supplement: Supplementary file 6 — Supplementary Figure S6-1 [file 41419_2019_1784_MOESM6_ESM.pdf]

Figure.S6

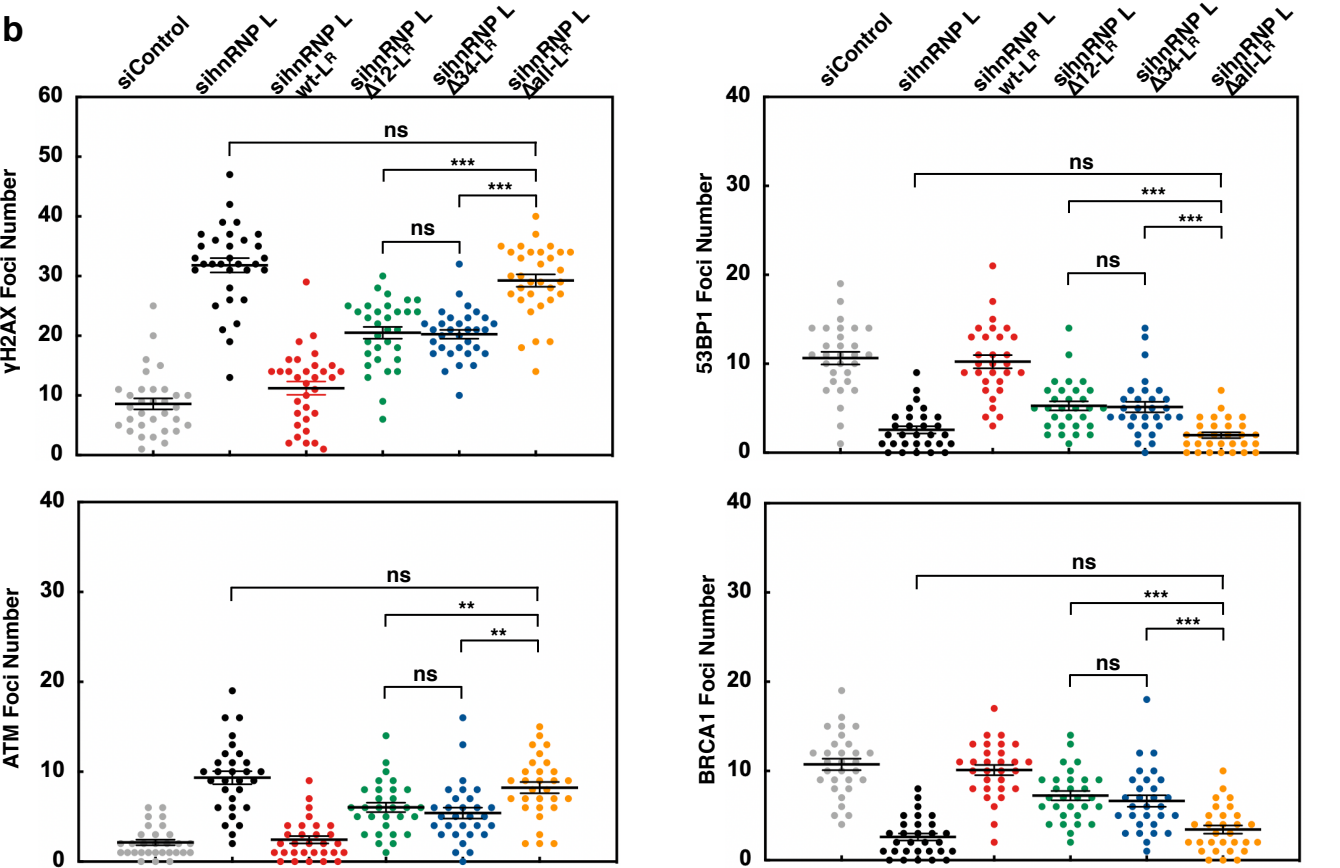

Supplement: Supplementary file 7 — Supplementary Figure S6-2 [file 41419_2019_1784_MOESM7_ESM.pdf]
