# Supplementary material for: Heterogeneous nuclear ribonucleoprotein L facilitates recruitment of 53BP1 and BRCA1 at the DNA break sites induced by oxaliplatin in colorectal cancer
Source: Cell Death Dis. 2019 Jul 18;10(8):550. doi: 10.1038/s41419-019-1784-x (PMC6639419; doi:10.1038/s41419-019-1784-x)
Supplement: Supplementary file 8 — Supplementary Table S1 [file 41419_2019_1784_MOESM8_ESM.docx]

**Table S1.** List of primers and siRNAs.

| **Related to** | **Name** | **Nucleotide sequence** |
| --- | --- | --- |
|  |  |  |
| **siRNAs Oligos** |  |  |
| Human | hnRNP L | GGAGCGUGAACAGUGUGCUUCUCUU |
| Human | 53BP1 #1 | GCCCAAGAGACACAGUACUCCUAUU |
| Human | 53BP1 #2 | CCCUAUGAAGCAGUAACACCUCUUA |
| Human | BRCA1 #1 | GGGCUAUCCUCUCAGAGUGACAUUU |
| Human | BRCA1 #2 | CAGAGGACAAUGGCUUCCAUGCAAU |
| Mouse | AID | GAGAUGCAUUUCGUAUGUUGGGAUU |
| Mouse | hnRNP L | CCUGGGAGAUCAUCCCGCAGAAUAU |
|  | H2AX-F | GGCCTCCAGTTCCCAGTG |
|  | H2AX-R | TCAGCGGTGAGGTACTCCAG |
|  | 53BP1-F | AGGTGGGTGTTCTTTGGCTTCC |
|  | 53BP1-R | TTGGTGTTGAGGCTTGTGGTGATAC |
| **qPCR Primers** | ATM-F | TGCTGACAATCATCACCAAGTTC |
|  | ATM-R | TCTCCCTTCGTGTCCTGGAA |
|  | BRCA1-F | ACAGCTGTGTGGTGCTTCTGTG |
|  | BRCA1-R | CATTGTCCTCTGTCCAGGCATC |
|  | 18s-F | TAGAGTGTTCAAAGCAGGCCC |
|  | 18s-R | CCAACAAAATAGAACCGCGGT |
| **PCR Primer** | G1 | CGGGTTCTGCGCCGCCATGT |
|  | G2 | CGTGGTGATCGAATAAATGGGGTTCA |
|  | Iμ-F | AAGGGCTTCTAAGCCAGTCC |
|  | Iμ-R | CACAACCATACATTCCCAGGT |
|  | Sμ-F | CAATGTGGTTTAATGAATTTGAAGTTGCCA |
| **γH2AX ChIP** | Sμ-R | TCTCACACTCACCTTGGATCTAAGCACTGT |
|  | Sα-F | TGAAAAGACTTTGGATGAAATGTGAACCAA |
|  | Sα-R | GATACTAGGTTGCATGGCTCCATTCACACA |
|  |  |  |
|  |  |  |
|  |  |  |
| **siRNA target site** | hnRNP L | NM_001005335, bases 566 to 590 |
| **modification** | Wild type | GGAGCGTGAACAGTGTGCTTCTCTT |
|  | Modified | GAAGTGTCAATTCTGTACTCCTATT |
