# Supplementary material for: Heterogeneous nuclear ribonucleoprotein L facilitates recruitment of 53BP1 and BRCA1 at the DNA break sites induced by oxaliplatin in colorectal cancer
Source: Cell Death Dis. 2019 Jul 18;10(8):550. doi: 10.1038/s41419-019-1784-x (PMC6639419; doi:10.1038/s41419-019-1784-x)
Supplement: Supplementary file 9 — Supplementary Table S2 [file 41419_2019_1784_MOESM9_ESM.docx]

**Table S2.** List of antibodies and cytokines.

| **Name** | **Company** | **Catalog Number** |
| --- | --- | --- |
| hnRNP L | Abcam | ab6106 |
| FLAG | Sigma | F1804 |
| IgM-PE | BD | 553409 |
| IgA-FITC | BD | 559354 |
| AID | eBioscience | # 14-5959-82 |
| ATM | Abcam | ab23420 |
| phospho-ATM | CST | #5883 |
| 53BP1 | CST | #4937 |
| phospho-53BP1 | CST | #2674 |
| BRCA1 | Abcam | ab16780 |
| phospho-BRCA1 | CST | #9009 |
| Ku80 | CST | #2180 |
| β-Actin | CST | # 3700 |
| phospho-H2A.X(Ser139) | CST | #9718S |
| phospho-H2A.X(Ser139), ChIP | Millipore | 05-636 |
| CD40L | eBioscience | #16-1541-82 |
| IL-4 | PeproTech | 214-14-20UG |
| TGF-β | Sino Biological | 80116-RNAH-5 |
