# Supplementary material for: Heterogeneous nuclear ribonucleoprotein L facilitates recruitment of 53BP1 and BRCA1 at the DNA break sites induced by oxaliplatin in colorectal cancer
Source: Cell Death Dis. 2019 Jul 18;10(8):550. doi: 10.1038/s41419-019-1784-x (PMC6639419; doi:10.1038/s41419-019-1784-x)
Supplement: Supplementary file 10 — Supplementary figure legends [file 41419_2019_1784_MOESM10_ESM.docx]

**Figure. S1 Generation of hnRNP L-defective SW620 cells by CRISPR/Cas9-mediated gene disruption.** **a** Schematic of Cas9/guide RNA (Cas9/gRNA) targeting sites in the hnRNP L genomic locus. Magenta boxs and grey dotted line represent coding and non-coding exons, respectively. The arrowheads (green) named with Target indicate the positions of Cas9/gRNA targeting; The arrows below the scheme show the positions of the PCR primers used in **b** (G1 and G2). **b** PCR (top) and Western blot (bottom) screenings of CRISPR knockout clones. **c** Schematic representation of the deleted regions in the hnRNP L alleles in the L22 clone. **d** PCR (top) and Western blot (bottom) screenings of CRISPR knockout clones from clone L22. **e** Western blot analyses of the hnRNP L-targeted clone L22.

**Figure. S2 Generation of hnRNP L-kockdown SW620 cells by shRNA-mediated expression disruption.** **a** Schematic of shRNA targeting sites in the hnRNP L exon locus. Magenta boxs and grey dotted line represent coding and non-coding exons, respectively. The arrowheads (red) named with Target indicate the positions of shRNA targeting. **b** Sequencing results of shRNAs inserted into the lentivirus vector. **c** SW620 cells transfected by shRNA shown at ×100 magnification under a fluorescence microscope. Scale bar represents 100 μm. **d** Cell proliferation assay. Graph showing proliferation of SW620 cells with shRNA-mediated hnRNP L disruption plotted over time. **e** Western blot analyses of the shRNA-mediated hnRNP L knockdown.

**Figure. S3 CRC cells are more sensitive to oxaliplatin in the absence of hnRNP L.** CRC cells at ×100 magnification under an inverted microscope. DMSO control; Scale bar represents 100 μm.

**Figure. S4 Quantitative RT-PCR (qRT-PCR) analysis of the critical DNA repair factors mRNA expression in the indicated colorectal cancer cell lines treated with siControl or sihnRNP L.** Data are presented as mean ± s.e.m of three experiments, ns: no significant difference, compared to siControl transfected cells, unpaired t test.

**Figure. S5 Deficiency of hnRNP L affects foci formation of DNA repair factors in response to treatment with oxaliplatin.** **a** Representative images of 53BP1, ATM and BRCA1 staining in human colorectal cancer cell line SW620 treated as indicated. The nuclei were stained with DAPI; **b** Scatter dot plots show the numbers of 53BP1, ATM and BRCA1 foci per nucleus. Approximately 35 to 45 nuclei were evaluated for foci formation for each sample, data are presented as mean ± s.e.m. ****P* < 0.001, unpaired t test.

**Figure. S6 The RRMs of hnRNP L is critical for recruitment of DNA repair factors at the DNA break sites induced by oxaliplatin.** **a** SW620 cells were treated as indicated and stained for γ-H2AX, 53BP1, ATM and BRCA1 in order to reveal the recruitment of these DNA repair factors to the DNA break sites. Scale bar represents 10 μm. **b** Scatter dot plots show the numbers of γ-H2AX, 53BP1, ATM and BRCA1 foci per nucleus. Approximately 30 to 35 nuclei were evaluated for foci formation for each sample, data are presented as mean ± s.e.m. ***P* < 0.01, ****P* < 0.001, unpaired t test.
